# Data Reduction Approaches for Dissecting Transcriptional Effects on Metabolism

**DOI:** 10.3389/fpls.2018.00538

**Published:** 2018-04-20

**Authors:** Kevin Schwahn, Zoran Nikoloski

**Affiliations:** ^1^Systems Biology and Mathematical Modelling Group, Max Placnk Institute of Molecular Plant Physiology, Potsdam, Germany; ^2^Bioinformatics Group, Institute of Biochemistry and Biology, University of Potsdam, Potsdam, Germany; ^3^Center of Plant Systems Biology and Biotechnology, Plovdiv, Bulgaria

**Keywords:** *E. coli*, *S. cerevisiae*, *A. thaliana*, partial correlation, principal component analysis, metabolomics, data reduction, regulation

## Abstract

The availability of high-throughput data from transcriptomics and metabolomics technologies provides the opportunity to characterize the transcriptional effects on metabolism. Here we propose and evaluate two computational approaches rooted in data reduction techniques to identify and categorize transcriptional effects on metabolism by combining data on gene expression and metabolite levels. The approaches determine the partial correlation between two metabolite data profiles upon control of given principal components extracted from transcriptomics data profiles. Therefore, they allow us to investigate both data types with all features simultaneously without doing preselection of genes. The proposed approaches allow us to categorize the relation between pairs of metabolites as being under transcriptional or post-transcriptional regulation. The resulting classification is compared to existing literature and accumulated evidence about regulatory mechanism of reactions and pathways in the cases of *Escherichia coli, Saccharomycies cerevisiae*, and *Arabidopsis thaliana*.

## 1. Introduction

Metabolism is the integrated output of transcription, post-transcriptional processes, translation and post-translational processes, and reflects the environment and changes in the availability of nutrients (Stitt, 2013; Johnson et al., 2016). The combined outcome of the aforementioned processes is the metabolic state of the system, observed in its metabolite and enzyme levels as well as the resulting reaction rates. The rates of metabolic reactions, however, are difficult to monitor and require involved computational integration of data and models (Tang et al., 2009; Sims et al., 2013). With advances in high-throughput techniques for monitoring of both metabolite and gene expression levels, the biological community is faced with the challenge of evaluating and integrating the obtained large-scale data to address several pressing questions: (i) which parts of metabolism are under regulation from transcriptional and downstream processes? (Less and Galili, 2008; Moxley et al., 2009; Haverkorn van Rijsewijk et al., 2011), (ii) how metabolism feeds back to transcription to coordinate the systemic functions? (Pego et al., 2000; Kresnowati et al., 2006; Ladurner, 2006; Lu et al., 2007; Kochanowski et al., 2017), and (iii) how and why are the changes at different cellular layers, like transcription and metabolism, suppressed or propagated to other layers? (Price et al., 2004; Gonçalves et al., 2017; Ledezma-Tejeida et al., 2017). In this context, we ask to what extent purely statistical techniques can be used to investigate whether data from metabolomics platforms in combination with transcriptomics data corroborate existing findings or yield new insights into transcriptional control of metabolism.

Microarray and RNA-sequencing techniques can measure several thousand genes from multiple conditions and time points simultaneously (Meyers et al., 2004; Jain, 2012). In contrast, metabolomics platforms provide measurements for only a fraction of the metabolon, including all metabolites in a given system (Fernie et al., 2004). Despite the growing number of publically available data sets, the case in which both data types are available from the same experiments is limited to only few observations (i.e., experiments, time points, replicates). Therefore, any method which is used to jointly investigate transcriptomic and metabolomic data faces the problem of high dimensionality of both data types and difference in the number of components measured. As a result, various multivariate statistics approaches have been evaluated to facilitate the analysis of transcriptomic and metabolomic data from the same experiments.

Whatever the multivariate statistical approach used, its aim is to identify an association between one or more genes and one or more metabolites. As a result, we can classify the methods into those which establish an association between (i) single gene and single metabolite, (ii) multiple genes and a single metabolite, (iii) single gene and multiple metabolites, and (iv) multiple genes and multiple metabolites. The first set of approaches is the simplest and aims at identifying the association for a pair of gene and a metabolite (Tohge et al., 2015; Cavill et al., 2016) by applying different similarity measures, such as: Pearson and Spearman correlation (Urbanczyk-Wochniak et al., 2003; Gibon et al., 2006; Hannah et al., 2010) or time-shifted correlations, in case when time-series data are analyzed (Walther et al., 2010; Takahashi et al., 2011). A general observation is that there is a high number of correlations between transcripts and metabolites, rendering it challenging to determine molecular/cellular mechanisms, and that one metabolite correlates to multiple transcripts, likely due to pleiotropic effects (Urbanczyk-Wochniak et al., 2003; Hannah et al., 2010). Further, the type of observed correlation (positive or negative) highly depends on the experimental condition. Along these lines, the resulting associations have also been analyzed with methods from network analysis (Bradley et al., 2008; Redestig and Costa, 2011). Approaches based on correlation networks have been employed for annotation of gene function given information about the compound class and structure of the metabolite (Tohge and Fernie, 2010, 2012).

In the case where association between multiple genes and one metabolite is to be identified one can use several approaches. For instance, classical regression techniques can be readily employed, with additional regularization to address the issue of high-dimensionality of the data (Auslander et al., 2016). On the other hand, dimension reduction techniques coupled with network analysis can be used to identify such associations: For instance, Inouye et al. (2010) identified modules of coexpressed genes on which they perform principal component analysis (PCA). The association (per Spearman correlation) between the first principal component and a given metabolic data profile is used as a means to determine which modules have influence on the metabolite level. While regression-based analysis is unbiased, in the sense that it can include all measured genes, it requires large data sets for estimation of the model coefficients. On the other hand, the identified modules based on correlation may be change if new data are analyzed indicating bias in the identified associations. In principal, by symmetry, these approaches can be used to identify and study associations between multiple metabolites and a single gene (Kochanowski et al., 2017).

The most involved cases are those where associations are to be established between multiple genes and multiple metabolites. In this case, there have been several approaches developed and used in the joint analysis of transcriptomics and metabolomics data sets: Partial Least Squares (PLS) and its extensions (Bylesjö et al., 2007) and Canonical correlation analysis (CCA) (Jozefczuk et al., 2010). PLS aims to find the relation between two matrices *X* and *Y* by estimating the direction in *X* that explains most of the variance in *Y* (Boulesteix and Strimmer, 2007). Due to its multivariate nature, PLS regression is difficult to interpret. The orthogonal projections to latent structures (OPLS) was designed to improve the interpretation of the regression. The approach allows to remove variation form *X*, which is uncorrelated (orthogonal) to *Y*. The advantage compared to PLS is twofold: first, the orthogonal part of *X* can be separately investigated and secondly and more important the removal of uncorrelated variation increases the interpretation (Trygg and Wold, 2002; el Bouhaddani et al., 2016). Given two data sets *X* and *Y*, CCA finds the canonical variates, *U* = *a*′*X* and *V* = *b*′*Y*, so that the correlation between *U* and *V* is maximized. The advantage of CCA is, that it is invariant with respect to transformation of the variables. However, the calculation of the CCA requires the inverse of *XX*^*T*^ which is challenging when the number of transcripts or metabolites exceeds the number of observations (as is the case for most biological studies). A solution to this dimensionality problem is to focus on a subset of the data, so that the number of transcripts (metabolites) is smaller than the number of observations, which may introduce bias in the analysis (Jozefczuk et al., 2010).

While the four classes of approaches can determine association between a subset of genes and a subset of metabolites, they cannot be used to determine if the relation between two metabolites is under transcriptional or post-transcriptional control. This question goes beyond the analysis of the affects of transcript on the level of metabolites, but rather on the coordination between metabolite levels. Addressing this issue will shed light on the transcriptional control of metabolic coordination. To this end, we propose two approaches rooted in a combination of partial correlation and dimension reduction techniques. We tested our proposed approaches with data sets from *E. coli, S. cerevisiae* and *A. thaliana* to identify metabolite pairs which are associated either by transcriptional or post-transcriptional regulatory effects. Our proposed approach might be used for biotechnology studies, where it can suggest metabolites whose relationship is under transcriptional regulation and is therefore easier to manipulate through genetic engineering.

## 2. Materials and methods

### 2.1. Data used in the study

The data used in this study were downloaded from the supplementary of Jozefczuk et al. (2010) and contain metabolomic and transcriptomic data from *E. coli* under different conditions (cold, heat, change from glucose to lactose and oxidative stress) as well as control treatment. The metabolomic data were generated by gas chromatography-mass spectroscopy (GC-MS) and contain 192 metabolites. All measured metabolites were normalized to cell number and the chromatographic internal standard. Transcript data were measured with microarray based technique and 4,440 transcripts were detected. The normalization procedure of the transcript data is described in the Materials and Methods section of Jozefczuk et al. (2010). In total 82 common data points were used for the analysis. Additionally, *A. thaliana* data from the study of Caldana et al. (2011) were used. The metabolomic data were generated by GC-MS and consists of 92 metabolites measured under the following conditions: 21°C at 75 μE *m*^−2^ *s*^−1^, 150 μE *m*^−2^ *s*^−1^ light intensity and darkness, 4°C at 85 μE *m*^−2^ *s*^−1^ light intensity and darkness, 32°C at 150 μE *m*^−2^ *s*^−1^ and darkness. Therefore, the analyzed data set consists of metabolic time series covering 20 time points and gathered under seven different light and temperature combinations. The normalization procedure of both data types is described in the Materials and Methods section of Caldana et al. (2011). Further, a data set from *S. cerevisiae* containing metabolomic and transcriptomic data from three different growth conditions, nitrogen upshift (shift from proline two glutamine), nitrogen downshift (shift from glutamine two proline) and Rapamycine treatment, was included. The data set contains 256 metabolites measured with FIA-QTOF-MS and 5,716 transcripts measured with Affymetrix chips (Oliveira et al., 2015). The normalization procedure is described in the Materials and Methods section of Oliveira et al. (2015) and the data were used as provided in the Supplementary. As the dimensions of the two data sets, i.e., transcripts and metabolites, need to agree, only matching time points per experiment were taken into account. The complete list of experiments and the appropriate time points is provided in Supplemental Table 1. In total 41 data points were used per metabolite and transcript.

### 2.2. PCA and partial correlation

PCA is a statistical procedure that uses an orthogonal linear transformation to convert a set of observations of possibly correlated variables into a set of values of linearly uncorrelated variables called PCs. The PCs are ordered according to the variance they explain (Wold et al., 1987).

Partial correlation measures the relationship (correlation) of two variables while controlling for a third or more variables. When using a single controlling factor, one calculates the first order partial correlation. If the number of controlling factors is higher, their information is recursively removed and the second or higher order partial correlation is determined. The zero order partial correlation is the same as the Pearson correlation. The expression for recursive calculation of partial correlation between the variables *X* and *Y*given a set of controlling variables in the set *V* is given by

(1) $$\begin{array}{r} r_{XY.V}=\frac{r_{XY.V/Z}-r_{XZ.V/Z}r_{YZ.V/Z}}{\sqrt{1-r_{XZ.V/Z}^{2}}\sqrt{1-r_{YZ.V/Z}^{2}}}. \end{array}$$

where *Z* ∈ *V*.

### 2.3. Combination of PCA and partial correlations to investigate influence of transcripts on metabolites

The combination of partial correlation and PCA allows the calculation of the two approaches Transcriptional dependent Partial Correlation (TPC) and Post-transcriptional dependent Partial Correlation (PPC).

We compute the first *p* PCs of the transcript data and use them as controlling variables for the partial correlation for each combination of metabolites. The number of PCs to choose was investigated based on the Broken-Stick model (Jackson, 1993), Kaiser-Guttman criteria (Yeomans and Golder, 1982) and the Horn's parallel analysis (PA) (Horn, 1965; Dinno, 2009). For the Broken-Stick model a distribution is calculated $\lambda_{i}=\sum_{k=i}^{p}\frac{1}{k}$, where *p* is the number of variables and λ_*i*_ the eigenvalue of the *ith* component (Jackson, 1993). In the Kaiser-Guttman approach, the PCs with an eigenvalue above the mean of the eigenvalues are regarded as significant (Yeomans and Golder, 1982). We performed Horn's parallel analysis by randomizing the transcript data and calculating the eigenvalues for the randomized data. A PC is identified as significant if its eigenvalue is larger than a chosen percentile of the distribution of eigenvalues of that component. We performed 1,000 randomization and regarded a PC as significant, if it exceeds the 99 percentile of the distribution of eigenvalues. We then compute significant differences of Pearson correlation and in-significant partial correlation pairs after removing the first *p* representative PCs from the transcriptomic data, yielding TPC. This gives transcriptionally regulated pairs of metabolites. In contrast we can use the same first *p* representative PCs to calculate the PPC, using the significant differences of Pearson correlation and significant partial correlations.

### 2.4. Calculating significant differences with permutation test

Testing for significant interactions of metabolites was performed by permutating the transcript and metabolite data component-wise. Calculations based on the two approaches are repeated for each of the 5,000 permutations. For all approaches we adjusted for multiple hypothesis testing, using Benjamini-Hochberg with a significance level α = 0.01.

### 2.5. Algorithm implementation

All analysis were performed in R (R-Core-Team, 2017) using the default functions and the corr.test() function of the psych package. For the recursive calculation of the partial correlation the pcor.rec() function was used, downloaded at http://www.yilab.gatech.edu/pcor.R. Evaluation of the permuted data to determine significance was implemented as stand-alone function. The estimation of the Kaiser-Guttman and the Broken-Stick model were done based on the provided function in the supplemental material of Borcard et al. (2011).

## 3. Results

### 3.1. Two novel methods for categorization of metabolite pairs based on transcriptional effects

**Figure 1:**
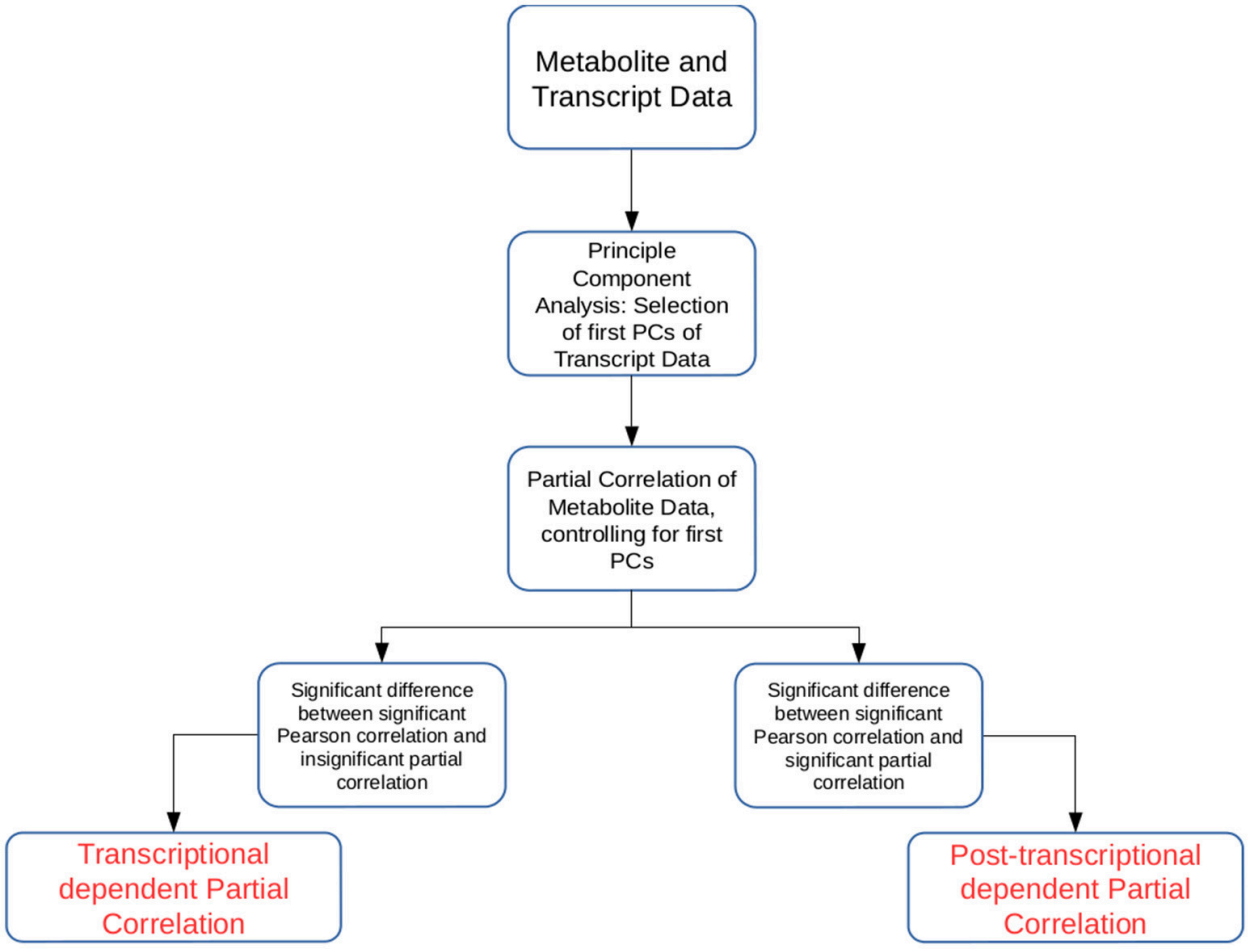
Schematic overview of the two approaches introduced in the study. The approaches Transcriptional dependent Partial Correlation (TPC) and Post-transcriptional dependent Partial Correlation (PPC) use the first *p* PCs of the transcriptomic data as control variables in the partial correlation calculation.

In this study we developed two approaches that allow the simultaneous investigation of transcriptomic and metabolomic data from the same experimental setup (see Figure 1). The novelty of the proposed approaches lies in the way the transcriptomic data are used to partial out (remove) the effect of the transcription layer from the metabolitc layer. Partial correlation has been used to investigate large-scale data sets from different *omics* technologies (de la Fuente et al., 2004; Veiga et al., 2007; Ursem et al., 2008; Wu et al., 2013). Partial correlation quantifies the association between two variables, while controlling for the influence of another set of variables. Therefore, it has been helpful in identifying non-spurrious associations (Baba et al., 2004). However, as higher order partial correlations are calculated iteratively, the calculation quickly becomes unfeasible with large transcriptomic or metabolomic data sets.

Our first approach, termed TPC, aims at identifying pairs of metabolites whose association is related to transcriptional regulation. The approach is composed of four steps: (1) We calculate the first *p* principal components (PCs) of the trancriptomic data, (2) we determine all metabolite pairs which have a significant Pearson correlation coefficient, (3) we determine all metabolite pairs which have non-significant partial correlation upon removal of the controlling variables, i.e., the *p* PCs from step (1), above, and (4) for the pairs of metabolites in the sets obtained from (2) and (3), we select those that show a significant difference between their Pearson correlation and partial correlation values. The reason for such construction of the approach is the following: if the removal of the significant PCs leads to a non-significant partial correlation between two metabolites, their association was due to transcriptional regulation. As the significant PCs capture most of the transcriptional effects, by finding the partial correlations we remove most of the transcriptional influence on the association between the two metabolites. The statistical tests ensure that the consideration of the significant PCs indeed break the significant association between metabolites and that the difference between the values is significant. To determine statistical significance we rely on permutation tests (see section Materials and Methods).

The second approach, termed PPC, follows a similar methodology. Again, the significant PCs from the transcriptomics data set are used as control variables in the partial correlation analysis for pairs of metabolites. In contrast to the TPC approach, we select those pairs of metabolites that are significantly associated upon removal of the significant PCs. Similar to TPC, we select those pairs of metabolites whose partial correlations show significant difference from the values of the respective Pearson correlation coefficient. The approach is based on the premise that if correlation remains upon removal of the transcriptional effect, the observed association is due to post-transcriptional regulation of the two metabolites. The significant difference to the observed Pearson correlation is employed to ensure that the observed partial correlation is due to post-transcriptional effects.

As both approaches relies on the estimation of principal components from the transcriptiomic data, the question arises, how many should be used for the analysis? More components will increase the computation time, while a to small number of PCs will not integrate sufficient transcriptomic information into the analysis. Multiple approaches have been reported to estimate the significant PCs. We employed the Kaiser-Guttman criteria (Yeomans and Golder, 1982), the Broken-Stick model (Jackson, 1993) and Horn's parallel analysis (PA) (Horn, 1965; Dinno, 2009) (see section Materials and Methods). Overall, we used our TPC and PPC approach on three different data sets, namely from *E. coli, S. cerevisiae* and *A. thaliana* (see section Materials and Methods). To this end, we investigated the number of significant PCs for each of the available data sets. The Kaiser-Guttman approach suggested the use of three PCs in each of the three data sets, whereas the Broken-Stick model suggest the usage of only one PC for *E. coli* and *A. thaliana* and two for *S. cerevisiae*. The PA approach confirms the one PC for *E. coli* and two for *S. cerevisiae*. However, the approach estimates two significant PCs for the *A. thaliana* data set. Overall, we found between one and three significant PCs, depending on the approach and data set (see Supplemental Figures 1–3). Therefore, we decided to use three PCs as a good compromise between the variance of the transcript data explained and the running time of the algorithm.

### 3.2. Transcriptional and post-transcriptional control of metabolite associations in *E. coli*

In this section, we applied our approaches with a transcriptomics and metabolomics data set from *E. coli* (see section Materials and Methods), containing the levels of 192 metabolites and 4,400 genes over five conditions. Employing the TPC resulted in 87 metabolite pairs under transcriptional control (Supplemental Table 2), whereas 630 metabolite pairs were found to be under post-transcriptional control with the PPC approach (Supplemental Table 3). As a first control, we did not identify an overlap between the pairs of metabolites detected with the two approaches.

**Figure 2:**
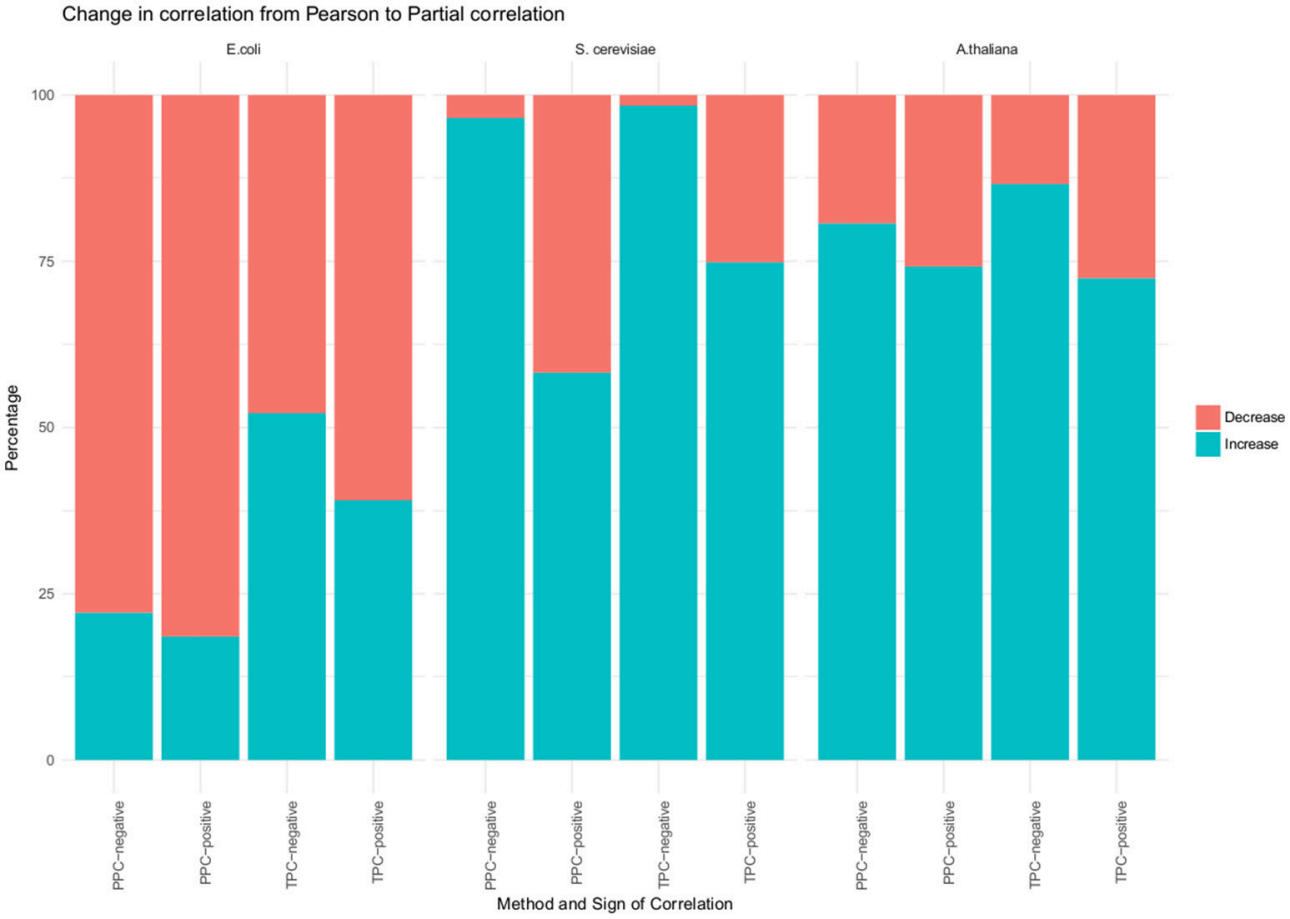
Changes from Pearson to partial correlation. Changes from Pearson to partial correlation for all three organism (*E. coli, S. cerevisiae* and *A. thaliana*) and for TPC, positve correlation; TPC, negative correlations; PPC, positive correlations; PPC, negative correlations. The blue portion of the bar represents the percentage of significant correlations whose absolute value increased from Pearson to partial correlation. The red portion of the bar represents the percentage of significant correlations whose absolute value decreased from Pearson to partial correlation.

In a first general investigation we found no change in the sign between Pearson and partial correlation. However, we investigated, if the absolute value of the correlation increased or decreased upon performing the partial correlation (Figure 2). Most of the significant correlations had a lower value when using partial correlation, in comparison to the Pearson correlation. More than 80% of the positive correlations found with the PPC approach decreased and around 60% of the positive correlations from the TPC approach. However, the overall observed differences between the values of Pearson and the partial correlation were between 0.005 and 0.03. Although, we did observe a change in the correlation with our approach, the magnitude is small.

In the following, we focused on the analyses of annotated metabolite pairs to allow for a comparison to the results previously reported in the literature. Out of the 87 metabolite pairs from the TPC approach 19 metabolite pairs (Supplemental Table 4) were unambiguously identified, whereas 132 of the 630 pairs of the PPC approach (Supplemental Table 5) were unambiguously identified. For instance, phosphate and maltose showed Pearson correlation of −0.26 and a partial correlation of −0.25. Both metabolites are part of the phosphoenolpyruvate dependent phosphotransferase system (PTS). The system consists of three enzymes performing the phosphate transport from PEP onto a carbohydrate. Maltose is one of the acceptors and belongs to the Glucose-class within the PTS. The three enzymes in the PTS are EI, Hpr and EII, which are encoded on the pts-operon, which itself is transcriptional regulated and induced through glucose. We therefore found a metabolite pair participating in a fully transcriptional regulated pathway (Postma et al., 1993; Tchieu et al., 2001). The weak negative correlation is explained by the fact that PEP acts a the main phsophate donor and we therefore capture not the complete active level of phosphates in this pathway. The negative correlation is explained by the reversibility of the system. While the sugar, here maltose, is only involved in one of the reactions within the PTS, the phosphate can be transferred between the three proteins (Deutscher et al., 2006). Therefore, an increase of maltose will have a delayed effect on the phosphate pool.

Further, we investigated the literature regarding GABA and L-ornithine showing a Pearson correlation of −0.30 and a partial correlation of −0.28. The negative correlation is related to the fact that the two metabolites are competing substrates for the same enzyme. The processing of one metabolite by the enzyme leads to an accumulation of the other substrate, which was shown in simulation studies (Schäuble et al., 2013). GABA and L-ornithine are connected via the enzyme 4-aminobutyrate aminotransferase, since it can use GABA and N-acetyl-L-ornithine as substrates (Lal et al., 2014) and N-acetyl-L-ornithine can be transformed into L-ornithine by one additional reaction. The enzyme, 4-aminobutyrate aminotransferase, is encoded by the gene gabT (Kurihara et al., 2010) and is activated by the regulatory protein cAMP receptor protein (CRP) (Metzner et al., 2004). CRP regulates gabTs activity mostly under stress conditions, more precisely at starvation. Further, regulatory mechanisms that influence the expression of gabT are the sigma factors sigmaS and sigma38 which are encoded by the gene RpoS (Joloba et al., 2004). Therefore, it is expected that the association between GABA and L-ornithine is transcriptionally regulated by CRP and RpoS.

Finally, we investigated the identified pair of 3PGA and aspartate. For these metabolites, the observed Pearson correlation was 0.28 and the partial correlation was 0.27. Jozefczuk et al. (2010) reported that in general 3PGA decreases under stress conditions, while only under cold stresses aspartate levels increase. The weak positive correlation is likely due to the fact that we investigated the correlation over multiple conditions (Bradley et al., 2008). Aspartate can be synthesized out of oxaloacetate, which additionally stands in exchange with 3PGA through PEP within the glycolysis. We therefore are able to identify two metabolites from the same pathway separated by two reactions, taking part in the glycolysis and the TCA cycle. Both pathways are partially regulated on the transcriptional level. For instance, the transcription factor Cra is involved in feedback and feed-forward regulation within these pathways (Shimizu, 2013). It is activating the transcription of the gene coding for the enzyme isocitrate dehydrogenase, which is an essential step for the transformation from citrate to all further downstream metabolites in the TCA cycle (Prost et al., 1999). Overall, the regulatory process will influence the pair of 3PGA and aspartate.

To come to a general conclusion, we investigated the available literature involving the metabolite pairs identified by the PPC approach. In comparison to the TPC approach, we frequently found amino acids within the pairs of the PPC approach (Supplemental Table 3). As amino acids are regulated through feedback inhibition by their loaded tRNAs (Sanchez and Demain, 2008), our approach captured the post-transcriptional regulation. For further validation, we investigated the literature regarding the two pairs of PEP-valine (Pearson correlation of −0.35 and partial correlation of −0.37) and PEP-leucine (Pearson correlation of −0.37 and partial correlation of −0.39). The negative correlation of PEP and the amino acids leucine and valine were previously reported in Szymanski et al. (2009) under stress conditions, which are comparable to the experimental conditions from our data sets. The PEP generating enzyme, the pyruvate kinase, is inhibited by fructose 1,6-bisphosphate and structural similar metabolites (Speranza et al., 1990). The synthesis of PEP is therefore under strong post-transcriptional regulation. Additional, the both mentioned amino acids are produced from pyruvate. Pyruvate is altered into PEP by a reversible reaction linking it further to post-transcriptional regulation. Further, valine and leucine share part of their synthesizing pathways. Valine is involved in a feedback inhibition of the enzyme acetohydroxy acid synthase and inhibits the leucine and the isoleucine synthesis as well. Furthermore, leucine inhibits its own producing enzymes (a-isopropylmalate synthase) regulating the group of amino acids coming from pyruvate. All three metabolites of the pairs are under post-transcriptional regulation. In addition, we found metabolites belonging to the TCA cycle and related reactions. Among these metabolites are malate, fumarate, PEP, 3PGA and GABA. Out of these malate and PEP (Pearson correlation of −0.29 and partial correlation of −0.31) were previously reported to be negatively correlated (Szymanski et al., 2009). PEP level increases under stress, while malate and precursors decrease. In contrast, the pair of 3PGA and GABA are positively correlated (Pearson correlation of 0.30 and partial correlation of 0.29). 3PGA level were reported to decreases under stress (Jozefczuk et al., 2010), while Szymanski et al. (2009) reported that amino acids decreased under stress conditions, which will effect GABA as well. The prevailing regulatory mechanism in the TCA cycle are product inhibition, substrate availability and competitive feedback inhibition. The citrate synthase is inhibited by citrate, further Succinyl-CoA is a competitor with acetyl-CoA for the citrate synthase as well. The first example is a product inhibition, whereas the second example is competitive feedback inhibition. Further, the isocitrate dehydrogenase is regulated by phosphorylation in *E. coli*. After phosphorylation the enzymes becomes inactive. Therefore, the TCA cycle is highly regulated on the post-transcriptional level (Voet and Voet, 2011). We can therefore confirm that malate, PEP, 3PGA and GABA are under post-transcriptional regulation.

Our approach allows to distinguish between metabolite pairs with associations controlled at transcriptional or post-transcriptional level. Therefore, we extended our analysis to data sets of *S. cerevisiae and A. thaliana*, aiming to reproduce the classification of metabolite pairs into transcriptional and post-transcriptional associated at higher organism.

### 3.3. Prevailing regulatory effects in *S. cerevisiae*—comparison with published results

So far we were able to identify the prevailing regulatory mechanism between identified pairs of metabolites. However, our comparison focused on a broad literature comparison, but did not compared our approach directly with a comparable method capable of integrating transcriptomic and metabolomic data into a combined analysis. Therefore, we chose to complement our study with a comparison with the results obtained in Oliveira et al. (2015). The study investigated the regulatory effect occurring during a nitrogen supply shift (upshift and downshift) as well as the treatment with Rapamycin in *S. cerevisiae*. Metabolite and transcript data were measured at up to 19 time points for the metabolite data and up to 8 time points for the transcript data for each of the three conditions. The overlapping eight time points are therefore ideal for our proposed method. In the original study, the authors used Bayesian inference to assign each metabolite to one of the four network motifs “unrelated” (no regulation related to TORC1), “downstream” (metabolites post-translational regulated downstream of TORC1), “upstream” (transcriptional regulation by metabolites upstream of TORC1) and “parallel” (transcriptional regulation by metabolites parallel of TORC1). The assignment of metabolites into on of the four categories is done by evaluating the dynamic dependence of metabolite and transcript pairs over time and the association of each metabolite with a specific set of genes regulated by TORC1, called “representation of TOR genes.” Both features are combined in a Bayesian inference framework approach to calculate the probability for each metabolite to belong to one of the four motifs. If the probability is above 50%, the metabolite is assigned to that particular motif. Eight metabolites were assigned to the downstream motif, eight metabolites to the upstream motif and eight metabolites to the parallel motif.

We used their provided data and applied our approaches, which resulted in 1,221 unique pairs with the TPC approach (Supplemental Table 6) and 4,239 unique pairs with the PPC approach (Supplemental Table 7). We compared the “downstream” assigned metabolites with our PPC approach, whereas the motifs “parallel” and “upstream” both relate to transcriptional regulation and were compared to our TPC approach. Similar to the results of *E. coli*, we observed no change in the sign of correlation between Pearson and partial correlation (Figure 2). In addition, we report the number of significant correlations above and below certain thresholds in Table 1. We observed higher significant correlations with the PPC approach for positive correlations, as well as for negative correlations, in comparison to the TPC approach. We found correlation above 0.9 with the PCC approach, whereas the correlations of the TPC did not exceed 0.65.

**Table 1:** Number of significant correlations above certain thresholds for the TPC and PPC approach for the data of Oliveira et al. (2015).

**Threshold**	**Number of significant TPC correlation**	**Number of significant PPC correlation**
> 0.9	0	4
> 0.8	0	151
> 0.7	0	567
> 0.6	2	1,170
> 0.5	43	2,152
> 0.4	456	3,260
< −0.4	49	495
< −0.5	1	125
< −0.6	0	16
< −0.7	0	0

Within the eight metabolites assigned in the “downstream” motif, we found 10 metabolite pairs with the PPC approach (see Table 2). Only trehalose-6phosphate and tetracosanoate are not part of any pair. Each of the remaining metabolites was part of at least two and up to four pairs. We found 16 metabolites of the “upstream” and “parallel” motifs, and we identified 11 pairs between these metabolites with the TPC approach (see Table 3). Only two pairs were found within the “parallel” group, the remaining nine pairs were between the groups “upstream” and “parallel.”

**Table 2:** Metabolite pairs found within the downstream motif of the approach by Oliveira et al. (2015) and our Post-transcriptional dependent Partial Correlation (PPC) approach.

**Metabolite 1**	**Metabolite 2**	**Pearson correlation**	**Partial correlation**
Pyrroline-3H-5C	Adenosine	0.484	0.433
Pyrroline-3H-5C	dGuanosine	0.483	0.433
Pyrroline-3H-5C	IMP	0.759	0.736
Indole-3-acetate	Adenosine	0.584	0.538
Indole-3-acetate	dGuanosine	0.584	0.538
Indole-3-acetate	IMP	0.616	0.571
Adenosine	IMP	0.744	0.708
Adenosine	L-Aspartate	−0.471	−0.418
dGuanosine	L-Aspartate	−0.471	−0.418
dGuanosine	IMP	0.744	0.701

**Table 3:** Metabolite pairs found within the upstream and parallel motif of the approach by Oliveira et al. (2015) and our Transcriptional dependent Partial Correlation (TPC) approach.

**Metabolite 1**	**Metabolite 2**	**Pearson correlation**	**Partial correlation**
NAD	AICAR	0.468	0.508
Thiamin triphosphate	AICAR	0.468	0.392
Thiamin triphosphate	L-Leucine	0.367	0.397
Thiamin triphosphate	5-L-Glutamyl-L-alanine	0.452	0.398
Ornithine	Dihydroxyacetone	−0.415	−0.377
Ornithine	Glyceraldehyde	−0.415	−0.377
Ornithine	D-Lactate	−0.415	−0.377
Ornithine	Imidazole glycerol-P	−0.374	−0.289
L-Leucine	AICAR	0.434	0.462
GABA	Glyceraldehyde	−0.384	−0.348
GABA	Glutamine	−0.388	−0.319

Overall, our approaches were able to categorize all investigated metabolites into transcriptionally or post-transcriptionally associated. In contrast, in the study of Oliveira et al. (2015) the majority of metabolites were assigned to the “unrelated” motif or none. The main reason is that their study focuses on TORC1 dependent regulation, while our approaches integrate all regulatory effects given the available data sets. We can therefore give a comprehensive overview of the regulatory mechanism affecting the associtions in which each metabolite is involved.

### 3.4. Transcriptional control of metabolite associations in *A. thaliana*

We also investigated a data set from the model plant *Arabidopsis thaliana* containing the levels of 92 metabolites and 15,089 genes over 7 conditions (see section Materials and Methods). Within this data set, we found 295 transcriptional associated metabolite pairs with the TPC approach (Supplemental Table 8). The PPC approach yield in total 1,534 metabolite pairs under post-transcriptional control (Supplemental Table 9). Similar to the results of the two previous investigated data sets, we did not observe a change in the sign of the correlation from Pearson to partial correlation. In contrast to *E. coli*, we observe metabolite pairs with a higher absolute partial correlation value than Pearson correlation value (Figure 2). We found more than 72% of the positively correlated metabolite pairs identified with TPC, more than 74% of the positively correlated metabolite pairs identified with PPC and 80% of the negatively correlated metabolite pairs identified PPC have a higher absolute partial correlation, than the respective Pearson correlation. However, the magnitude of the changes is in the range of 0.01–0.08, similar to the observations from the *E. coli* data set.

Like in the analysis of the *E. coli* data set, we focused on a subset of fully annotated metabolite pairs. Of the 295 metabolite pairs of the TPC approach 150 were unambiguous annotated (Supplemental Table 10), whereas 773 out of the 1,534 metabolite pairs of the PPC approach were unambiguous annotated (Supplemental Table 11). The difference in numbers of the TPC and PPC approach indicate a general tendency in the regulation toward post-transciptional regulation. This was already noted in the results of the original study in which the authors observed only a minor interconnection of the measured metabolites and transcripts. Further, they assume that this would change with a higher proportion of secondary metabolites, as primary metabolites have to react faster during external changes and are therefore mostly under post-transcriptional regulation (Caldana et al., 2011). We next focus on specific examples of both approaches to show their capability to distinguish between both regulatory mechanisms.

We start the investigation with the unique metabolite pairs identified with the TPC approach. We observed that the highest positive correlations are between amino acids and glycerol. In studies relate to heat stress and heat tolerance it was shown that glycerol increased as a response to heat. Additionally, the studies showed an increase of amino acids as alanine, beta-alanine, leucine, isoleucine and aspartate (Kaplan et al., 2004). We could report the pairs glycerol and isoleucine (Pearson correlation of 0.60 and partial correlation of 0.60), glycerol and leucine (Pearson correlation of 0.59 and partial correlation of 0.60) and glycerine and beta-alanine (Pearson correlation of 0.32 and partial correlation of 0.33). The measurements were done under different light and temperature conditions, including highlight and high temperatures. It is therefore realistic to assume, that we observe a mild heat stress reactions. The regulation of heat stress response is reported to be completely under transcriptional regulation (Ohama et al., 2016), which agrees with our findings.

Within the results of the PPC approach, we found amino acids correlating with each other. This observation is in agreement with previously published results, showing that the synthesizing pathways of most amino acids are under post-transcriptional regulation, more precisely under allosteric product inhibition (Less and Galili, 2008). A well studied example is the branched-chain amino acid metabolism (BCAA), in which leucine, valine and isoleucine are synthesized. Each of these amino acids is reported several times within our PPC approach and forms pairs with other amino acids. Leucine and isoleucine are positively correlated to ornithine which is of interest as as ornithine is a precursor of glutamte. Glutamate is involved in the synthesis of the BCAA amino acids. The reactions involved in these amino acid synthesis pathways are reported be allosterically regulated (Binder, 2010).

Additionally, we found a relationship between skikimate and related amino acids, as well as shikimate and sugars. Shikimate is a precursor to the amino acids tyrosine, phenylalanine and tryptophan. Shikimate is negatively correlated to pheylalanine (Pearson correlation of −0.42 and partial correlation of −0.39) and tyrosine (Pearson correlation of −0.59 and partial correlation of −0.57). Tryptophan was not reported within the uniquely identified metabolite pairs. At the same time shikimate is positively correlated to pyruvic acid (Pearson correlation of 0.67 and partial correlation of 0.64), fructose (Pearson correlation of 0.74 and partial correlation of 0.76), glucose (Pearson correlation of 0.78 and partial correlation of 0.80) and sucrose (Pearson correlation of 0.74 and partial correlation of 0.73). We therefore observed that metabolites upstream of shikimate (sugars) were positiveley correlated, while downstream metabolites were negatively correlated. The pathway is partly feedback regulated meaning that the end products (amino acids) inhibit their production which explains the negative correlation. The sugars were positively correlated to shikimate as they are potential precursors (Tzin and Galili, 2010a,b).

In comparison *E. coli* we found more pairs with both approaches. The correlations of the TPC approach was higher than in *E. coli*. A similar situation was observed for the PPC approach. We observed more sugars and sugar derivatives in *E. coli*, whereas amino acids were mostly found with high positive correlation.

## 4. Discussion

In this study we proposed two approaches for a combined investigation of metabolic and transcriptomic data. The two proposed approaches are based on the concept of removing transcriptional information from metabolomic data, allowing us to categorize pairs of metabolites into transcriptional or post-transcriptionally regulated. The developed approach TPC allows the identification of transcriptionally regulated metabolites through a modified partial correlation approach using PCs of the transcriptomic data as controlling variables. The second approach, PPC, is based on a similar concept and it allows the identification of post-transcriptional regulation between pairs of metabolites.

The commonality of the investigate data sets is their focus on the change of central metabolites after perturbation or changing environmental conditions. It has been shown that in microorganisms the majority of primary metabolites are mainly regulated on the enzymatic level through feedback inhibition (Sanchez and Demain, 2008). Further, the post-transcriptional regulation allows the organism to react faster to changes in the environment (Caldana et al., 2011). The combination of these two criteria explain the larger amount of metabolite pairs found with the PPC approach, in comparison of the TPC approach. The low coverage of correctly annotated metabolites in the data sets restricted our analysis to a smaller subset of metabolites. Nevertheless, the annotated metabolites were sufficient to obtain an overview over the potential of the approaches. We demonstrated that there is experimental evidence in the literature that the proposed approaches are capable of detecting differences in the association of metabolites, namely if the association is due to transcriptional or post-transcriptional effects. Moreover, we could show that our results agree with the findings from the study of Oliveira et al. (2015). Metabolites that were reported to be post-transcriptionally regulated were also identified to participate in relationships identified by our PPC approach. We observed a similar situation with the transcriptionally associated metabolites, although we had to pool the reported metabolites from the “upstream” and “parallel” motif, as the TPC approach takes all transcriptional regulation mechanism into account.

**Figure 3:**
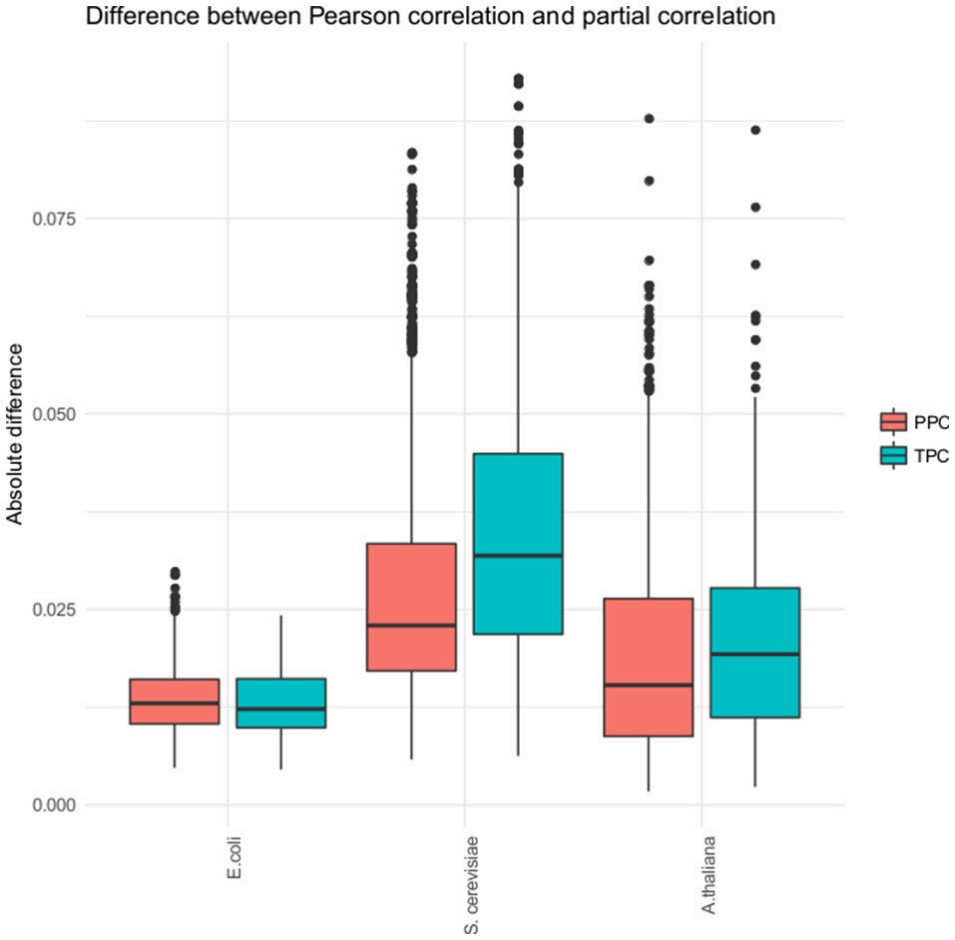
Distribution of the absolute difference of Pearson and partial correlation. The boxplots show the absolute difference of the Pearson and partial correlations for each of the three organism (*E. coli, S. cerevisiae*, and *A. thaliana*) and the two approaches, respectively.

While we observed a differentiation into pairs found by TPC and PPC, the detected partial correlation in each approach did not differ strongly from the found Pearson correlation (see Figure 3). The Pearson correlation captures most of the association already. Therefore, our approach does not strongly affects the correlation, but is a tool for categorizing the associations between metabolites. This claim is supported through two findings, the lack of overlap of metabolite pairs found with the two approaches in all three data sets and the low difference of the Pearson correlation and partial correlation for the identified metabolite pairs.

In the recent work of Bradley et al. (2008), they reported that the correlation between metabolites and transcripts depends on the experimental condition. The authors report that nearly no correlation was found when the correlation was investigated over multiple conditions, whereas high (positive or negative) correlations were observed if the conditions were investigated separately. Our approach aims at identifying the general underlying relations between metabolites and if these originate from transcriptional regulation or post-transcriptional regulation. While the magnitude of the correlation is often of interest for many studies, our approach allows to gain further knowledge through the classification of the identified metabolite associations. Employing the approaches over multiple conditions allows us to give a general statement about the regulation associating pairs of metabolites.

A potential application for our proposed approaches is metabolic engineering. Metabolic engineering aims at enhancing certain important pathways which leads to an overproduction of a metabolite of interest (Bailey, 1991; Nevoigt, 2008). A frequently employed technique is the over expression of genes associated with the metabolic pathway of interest. This technique has the disadvantage that the resulting phenotype (metabolite production) is difficult to predict and needs a strict monitoring for the validation. The results of the over-expression approaches might fall behind the expected yields of the metabolites. This shortcoming may be due to post-transcriptional regulation within the engineered pathway. Our method allows to investigate metabolic pathways before establishing over-expression lines and selecting metabolites and corresponding pathways which are mostly under transcriptional regulation, rather than post-transcriptional. This would allow biologists to focus their experiments to a smaller set of over-expression lines which would save both time and experimental resources.

Overall, we present here two approaches named TPC and PPC for investigating the prevalent regulatory mechanism of metabolite pairs. To our knowledge it is the first time that partial correlation is used to remove all transcriptional information from a metabolomic data set, removing not just the effect of a set of genes, but the majority of transcriptional regulation. This novel investigation methods will help to elucidate the complex regulatory mechanism of metabolites while employing well known and established statistical methods.

## Author contributions

ZN conceived the project and wrote the article with contribution of KS. KS performed the analysis and analyzed the data.

### Conflict of interest statement

The authors declare that the research was conducted in the absence of any commercial or financial relationships that could be construed as a potential conflict of interest.
