# Supplementary figures and images for: Data Reduction Approaches for Dissecting Transcriptional Effects on Metabolism
Source: Front Plant Sci. 2018 Apr 20;9:538. doi: 10.3389/fpls.2018.00538 (PMC5920133; doi:10.3389/fpls.2018.00538)

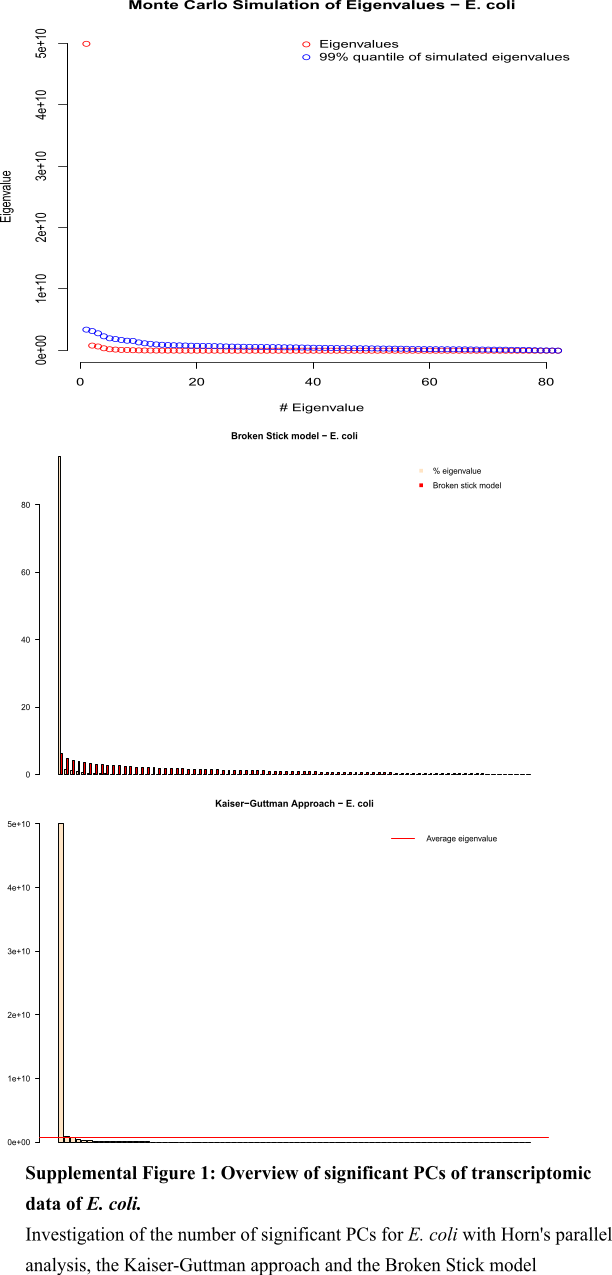

Supplement: Supplementary file 3 [file Image1.TIF]

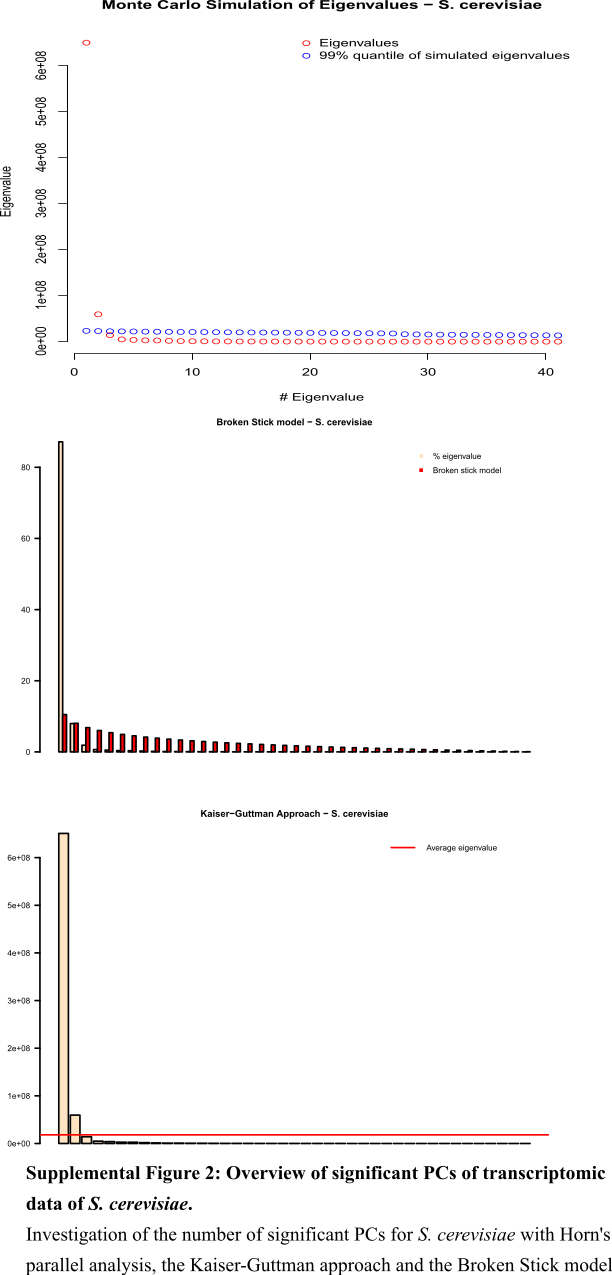

Supplement: Supplementary file 4 [file Image2.TIF]

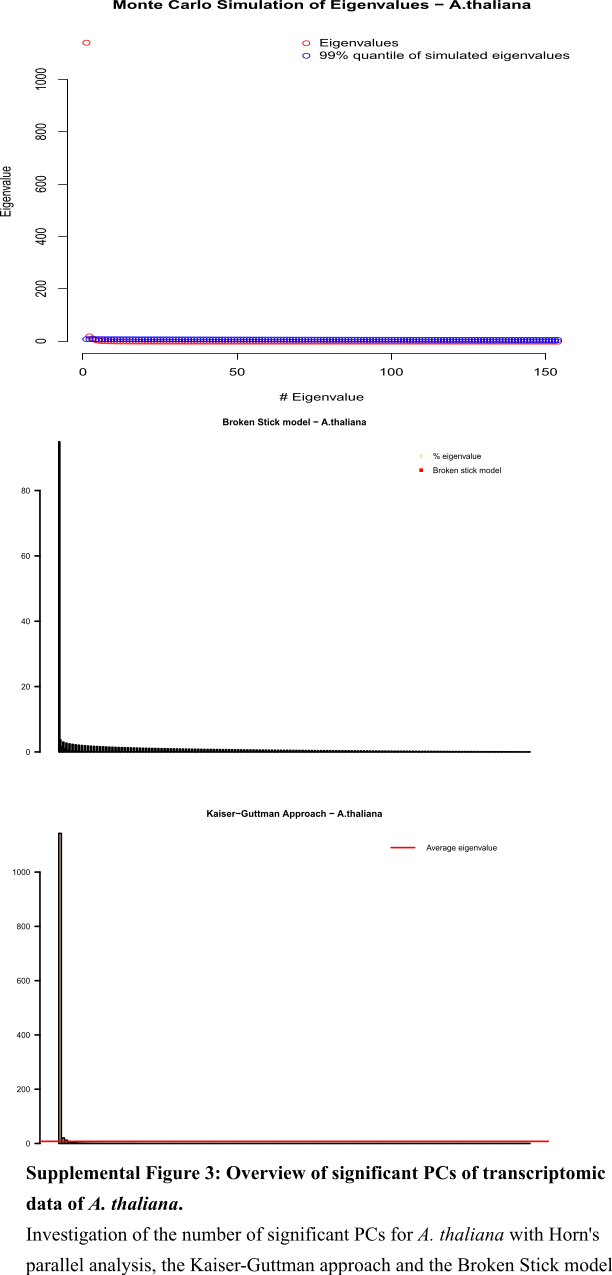

Supplement: Supplementary file 5 [file Image3.TIF]
